# Transmission of the First Influenza A(H1N1)pdm09 Pandemic Wave in Australia Was Driven by Undetected Infections: Pandemic Response Implications

**DOI:** 10.1371/journal.pone.0144331

**Published:** 2015-12-21

**Authors:** James E. Fielding, Heath A. Kelly, Kathryn Glass

**Affiliations:** 1 Victorian Infectious Diseases Reference Laboratory, North Melbourne, Victoria, Australia; 2 National Centre for Epidemiology and Population Health, The Australian National University, Canberra, Australian Capital Territory, Australia; 3 Centre for Epidemiology and Biostatistics, Melbourne School of Population and Global Health, The University of Melbourne, Parkville, Victoria, Australia; National Institutes of Health, UNITED STATES

## Abstract

**Background:**

During the first wave of influenza A(H1N1)pdm09 in Victoria, Australia the rapid increase in notified cases and the high proportion with relatively mild symptoms suggested that community transmission was established before cases were identified. This lead to the hypothesis that those with low-level infections were the main drivers of the pandemic.

**Methods:**

A deterministic susceptible-infected-recovered model was constructed to describe the first pandemic wave in a population structured by disease severity levels of asymptomatic, low-level symptoms, moderate symptoms and severe symptoms requiring hospitalisation. The model incorporated mixing, infectivity and duration of infectiousness parameters to calculate subgroup-specific reproduction numbers for each severity level.

**Results:**

With stratum-specific effective reproduction numbers of 1.82 and 1.32 respectively, those with low-level symptoms, and those with asymptomatic infections were responsible for most of the transmission. The effective reproduction numbers for infections resulting in moderate symptoms and hospitalisation were less than one. Sensitivity analyses confirmed the importance of parameters relating to asymptomatic individuals and those with low-level symptoms.

**Conclusions:**

Transmission of influenza A(H1N1)pdm09 was largely driven by those invisible to the health system. This has implications for control measures–such as distribution of antivirals to cases and contacts and quarantine/isolation–that rely on detection of infected cases. Pandemic plans need to incorporate milder scenarios, with a graded approach to implementation of control measures.

## Introduction

Influenza A(H1N1)pdm09 was identified in the United States and Mexico in April 2009 and spread rapidly around the globe [1, 2]. In temperate countries of the northern hemisphere, the pandemic strain emerged outside of the cooler months during which seasonal influenza epidemics typically occur, resulting in a first pandemic wave of moderate magnitude followed by a larger second in-season wave [3, 4]. In contrast, both waves in temperate southern hemisphere countries occurred in-season, with a considerably lower overall cumulative incidence of symptomatic infection and impact in terms of severe illness in the second wave [5].

Although Australia’s first case was reported in Queensland on 9 May, the second reported case in Victoria 11 days later was followed by a rapid increase in notified cases that was not observed in other states or territories [6, 7]. As the pandemic response progressed it became evident that despite the large number of notified cases, a high proportion had relatively mild symptoms and much lower case fatality risk compared to previous pandemics [8]. Influenza-like illness activity and proportion of influenza tests positive as measured by other surveillance systems was also moderate compared to other influenza seasons [9, 10]. Furthermore, there was a suggestion, supported by modelling, that community transmission of influenza A(H1N1)pdm09 in Victoria was well established before cases were identified [11].

These observations lead to the hypothesis that those with asymptomatic or clinically mild infections were driving the spread of the pandemic. To investigate this hypothesis, we developed a deterministic mathematical model to estimate the relative importance of different levels of disease severity in transmission of the first pandemic wave of influenza A(H1N1)pdm09 virus. We used data from observational studies to parameterise the model using the Australian population as an example.

## Methods

### Model structure

**Fig 1:**
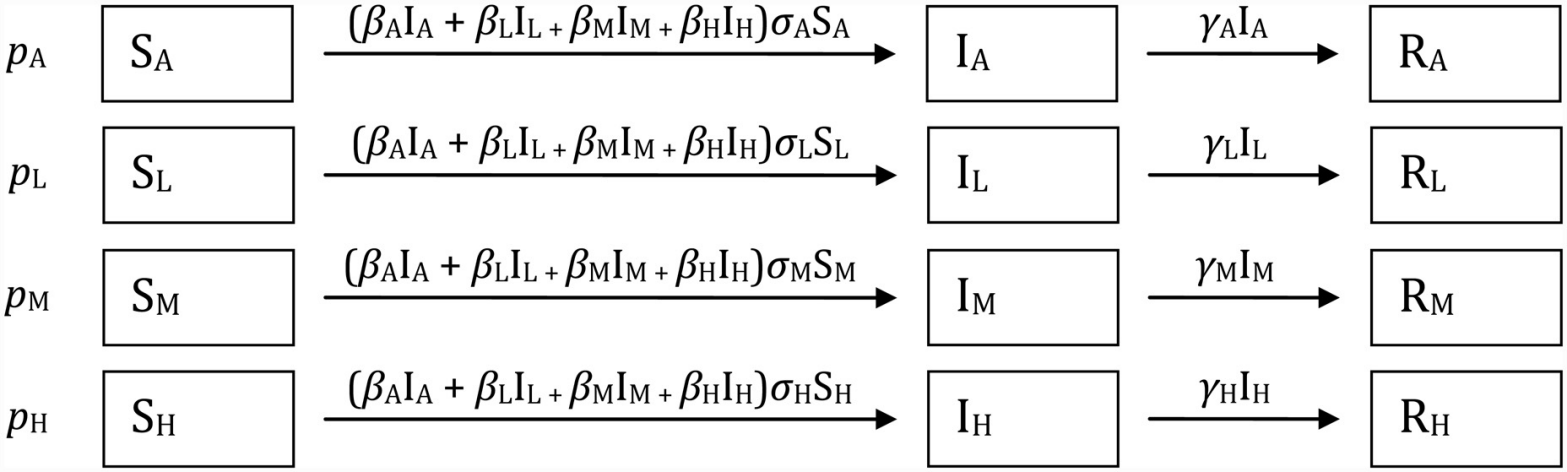
Influenza model with four levels of infection severity: asymptomatic (A), low-level symptoms (L), moderate symptoms (M) and hospitalised (H).

A deterministic susceptible-infected-recovered (SIR) model was constructed to describe the first wave of influenza A(H1N1)pdm09 transmission in a population structured by severity of infection. Four levels of infection severity were defined in the model: asymptomatic; low-level symptoms; moderate symptoms; and hospitalisation required, denoted by the subscript letters ‘A’, ‘L’, ‘M’ and ‘H’ respectively (Fig 1). Based on published outcome data and detailed further below, the population prior to the first wave of infection was proportionally assigned to four infection severity compartments of susceptible individuals (S). This stratification of the susceptible population assumed that the disease course was defined before infection by multiple determinants of infection severity, including underlying health status and immunity from prior infection and/or vaccination. Given limited data to parameterise differences in susceptibility by severity strata, our default model assumes that all infection severity groups had the same susceptibility and thus the same infection pressure acting on them. We therefore included severity stratum-specific susceptibility parameters (*σ*_i_) set to 1 in the baseline model, and subsequently tested the sensitivity of our findings to this assumption.

Each transmission rate *β*_i_ is a product of mixing rates (*μ*_i_) and a common fitting coefficient *θ*, while susceptibility (*σ*_i_) and duration of infectivity (1/*γ*_i_) varies by infection severity. The initial proportions of the population in each severity level is given by *p*i, where *p*_L_ = (1 –*q*).[1 –(*p*_A_ + *p*_H_)] and *p*_M_ = *q*.[1 –(*p*_A_ + *p*_H_)], and *q* is the proportion of symptomatic community cases that have moderate symptoms, and so are unable to undertake normal duties for two or more days.

Multiple studies have found no difference between viral loads and clinical severity, ranging from asymptomatic infection to acute respiratory distress syndrome [12–19]. We therefore assumed all severity classes were equally infectious (although duration of infectivity varied). Given these assumptions, the transmission parameter *β*_*i*_ that determines the infection rate from severity stratum *i* was calculated as the product of the strata-specific mixing parameters (*μ*_*i*_), and a common fitting coefficient *θ* as *β*_i_ = *θ*.*μ*_i_, where *i* is one of A, L, M or H. The fitting coefficient, *θ*, was defined in terms of the overall effective reproduction number, *R*_e_, as

$$R_{e}\;=\;\sum p_{i}.\frac{\beta_{i}}{\gamma_{i}}\;=\;\sum p_{i}.\frac{\theta \mu_{i}}{\gamma_{i}}$$

where *p*_i_ is the proportion in each severity stratum A, L, M or H, and 1/*γ*_i_ is the mean infectious period of an individual in stratum *i*. The equation can be rearranged to calculate *θ* by

$$\theta \;=\;R_{e}/\left(\sum p_{i}.\frac{\mu_{i}}{\gamma_{i}}\right)$$

Fig 1 provides a flow diagram for the model, where the force of infection *λ*, is given by *λ* = Σ *β*_i_.I_i_. By comprising the sum of each stratum-specific product of transmission parameter *β* and number of infected individuals I, the force of infection *λ* accounts for inter-group mixing (also see differential equations in S1 File). Given its emergence as a pandemic strain, the baseline model assumed a population susceptible to influenza A(H1N1)pdm09 with no previous immunity from vaccination or infection, and that re-infections did not occur in the timeframe considered. Later sensitivity analyses considered the implications of decreased susceptibility with increased infection severity through an example scenario of older individuals with some immunity and higher risk of hospitalisation.

### Selection of baseline parameters

Parameter descriptors, values and sources used in the model are summarised in Table 1. The proportional distribution of the susceptible population among the four infection severity compartments was estimated from published observational studies of influenza A(H1N1)pdm09 infections. The reported proportion of asymptomatic infections (*p*_A_) varied widely by study setting and population, but was estimated at 0.35 based on several household and school transmission studies [18, 20, 21]. Reported estimates of the hospitalised proportion (*p*_H_) were universally small at around 0.0025 [22, 23]. To divide the remaining 0.6475 proportion of symptomatic infections between cases with low-level and moderate symptoms, we used data from the New South Wales Population Health Survey which collected all-age community-level influenza-like illness (ILI) data across the state from July to September 2009 [24]. Of the survey participants reporting an ILI, an average of 76% were unable to undertake normal duties for two or more days (classified as moderate symptoms and denoted as ‘*q*’) and 24% (1 − *q*) were unable to undertake normal duties for at most one day because of their ILI (classified as low-level symptoms). Thus, 0.1554 and 0.4921 proportions of the susceptible population (*p*_L_ and *p*_M_) were assigned to the low-level and moderate symptoms compartments respectively.

**Table 1:** List of model parameters and their values.

Parameter	Notation*	Baseline value	Source(s)
Population proportion	*p*_A_, *p*_L_, *p*_M_, *p*_H_	0.35, 0.1554, 0.4921, 0.0025	[18, 20–24]
Proportion of symptomatic cases requiring ≥2 days off normal duties	*q*	0.76	[24]
Mixing coefficient	*μ*_A_, *μ*_L_, *μ*_M_, *μ*_H_	1.0, 0.9, 0.4, 0.1	
Recovery rate	*γ*_A_, *γ*_L_, *γ*_M_, *γ*_H_	1/3.2, 1/4.9, 1/4.9, 1/8.3	[25]

* Subscripts denote infection severity categories of asymptomatic (A), low-level symptoms (L), moderate symptoms (M) and hospitalised (H).

The relative mixing (or effective contact) parameters *μ* were defined as proportions relative to the asymptomatic class (*μ*_A_ = 1.0), with the level of mixing decreasing as infection severity increased. In the absence of published observational data, we used the NSW population health survey to inform our mixing assumptions for each severity stratum [24]. Given those with low-level symptoms were defined as being unable to undertake normal duties for at most one day because of illness, a slightly lower relative degree of mixing (*μ*_L_ = 0.9) was assumed. However, mixing was considered to be much lower for infections with moderate symptoms that prevented normal duties for two or more days (*μ*_M_ = 0.4) and mixing was lower still in those ill enough to require hospitalisation (*μ*_H_ = 0.1).

Studies have indicated heterogeneity in the duration of viral shedding between different severity classes. The parameters *γ*_i_ define the recovery rate in each severity category and are calculated as the inverse of the duration of infectiousness. Viral shedding duration was used as a proxy for duration of infectiousness, with values determined using weighted averages of medians from a systematic review of influenza A(H1N1)pdm09 virus shedding for asymptomatic, community-based and hospitalised cases [25]. The studies included in the review did not differentiate viral shedding of low level and moderate symptoms, thus the same weighted average of median duration from community-based cases was used for both these infection severity categories. As our focus here is on cumulative incidence and the relative contribution of each severity class to transmission, we do not model shedding dynamics in the individual, and assume a consistent level of viral shedding over the course of infection. Where this assumption may affect parameters, such as the mixing parameters *μ*_i_, we have conducted further sensitivity analyses.

### Model fitting and sensitivity analysis

MATLAB was used to simulate and calibrate the model using values of *R*_e_ within the limits of published estimates (range: 1.14–1.36) [26] that resulted in a total proportion of recovered individuals that was consistent with estimated age-standardised infection risks of 19% and 21% in two all-age studies in Australia and New Zealand respectively [23, 27]. The differential equations for the model are given in S1 File. Infection severity stratum-specific reproduction numbers were then calculated to determine the relative importance of each group in influenza A(H1N1)pdm09 virus transmission.

Sensitivity analyses were also undertaken in MATLAB to assess the relative influence of the disease severity proportions, mixing and recovery rate parameters on the risk of infection, with a fixed overall reproduction number. Given their dependence on q, the proportions of low-level and moderate symptoms parameters *p*_L_ and *p*_M_ were substituted with *q* in the sensitivity analysis. The mixing coefficient *μ*_A_ was also excluded from the sensitivity analysis because it is the reference value against which the other mixing parameters were compared. Triangular distributions of the ten parameter ranges (baseline value plus and minus 10%) were sampled 400 times using Latin hypercube sampling. Parameter outputs were then transformed into their ranks and partial rank correlation coefficients (PRCC) calculated, using the cumulative infection risk as the outcome. Parameters with a PRCC closer to -1 and +1 indicated a stronger impact on the model output, with the direction indicating a negative or positive correlation [28]. Note that as the focus of this analysis is to estimate the change in outcome across a range for each parameter, the sampling distribution has relatively little impact. We chose a triangular distribution for simplicity and because it is bounded, but tests with normal distributions gave similar results.

The results of the PRCC were also used to identify important parameters and test the effect of their variation, within plausible limits, on the infection severity stratum-specific reproduction numbers. In particular, we tested the effect of lowering *μ*_L_ from 0.9 to 0.7, increasing *μ*_M_ from 0.4 to 0.6, and increasing the duration of infectiousness for the moderate symptoms group (1/*γ*_M_). The *q* parameter was varied from a baseline value of 0.76 to 0.42, based on data from the Australian Flutracking surveillance system which provides weekly community-level ILI symptomatic infection risks not biased by health-seeking behaviour and clinician testing practices; in the 2011 and 2012 influenza seasons, an average of 42% of Flutracking participants reporting an ILI took two or more days off work or normal duties because of their illness [29]. The effect of lowering the proportion of asymptomatic cases (*p*_A_) from 0.35 to 0.13 (the average of three studies in Canada [15], Germany [17] and China [30]) was also tested.

We tested the sensitivity of our findings to the effect of those aged 65 years and older having higher pre-existing immunity to influenza A(H1N1)pdm09, but more likely to be hospitalised once infected. Susceptibility adjustments of 0.89 for the hospitalised stratum and 0.94 for the asymptomatic, low-level symptoms and moderate symptoms strata were calculated using pre-pandemic seroprevalence proportions of antibody to influenza A(H1N1)pdm09 of 0.06 and 0.33 for those aged less than 65 years and 65 years and older respectively [27], and that those aged 65 years and older comprised 15% of hospitalised influenza A(H1N1)pdm09 cases [31] and 0% of the other infection severity strata [32, 33].

## Results

**Fig 2:**
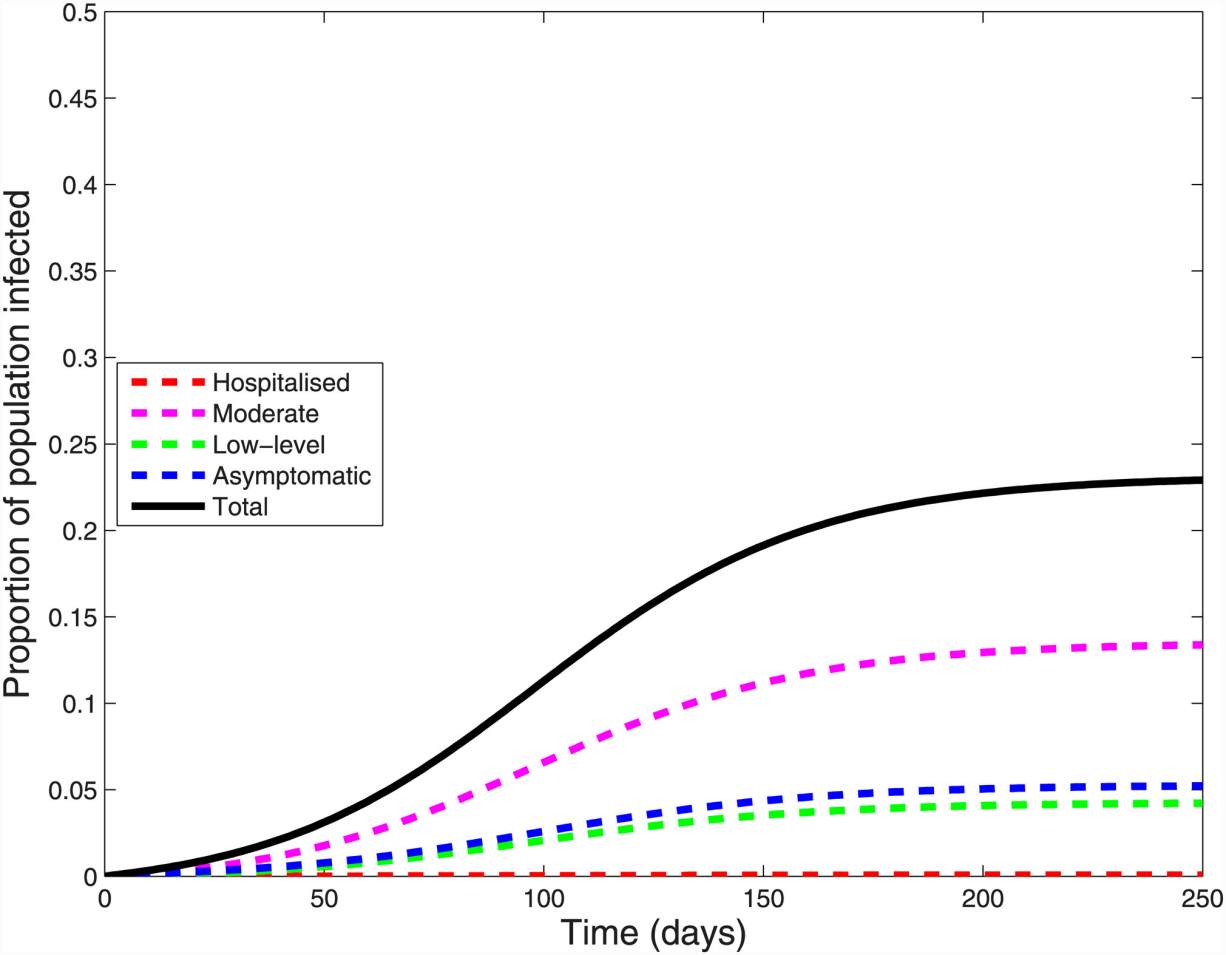
Cumulative incidence of influenza A(H1N1)pdm09 over time by infection severity.

**Fig 3:**
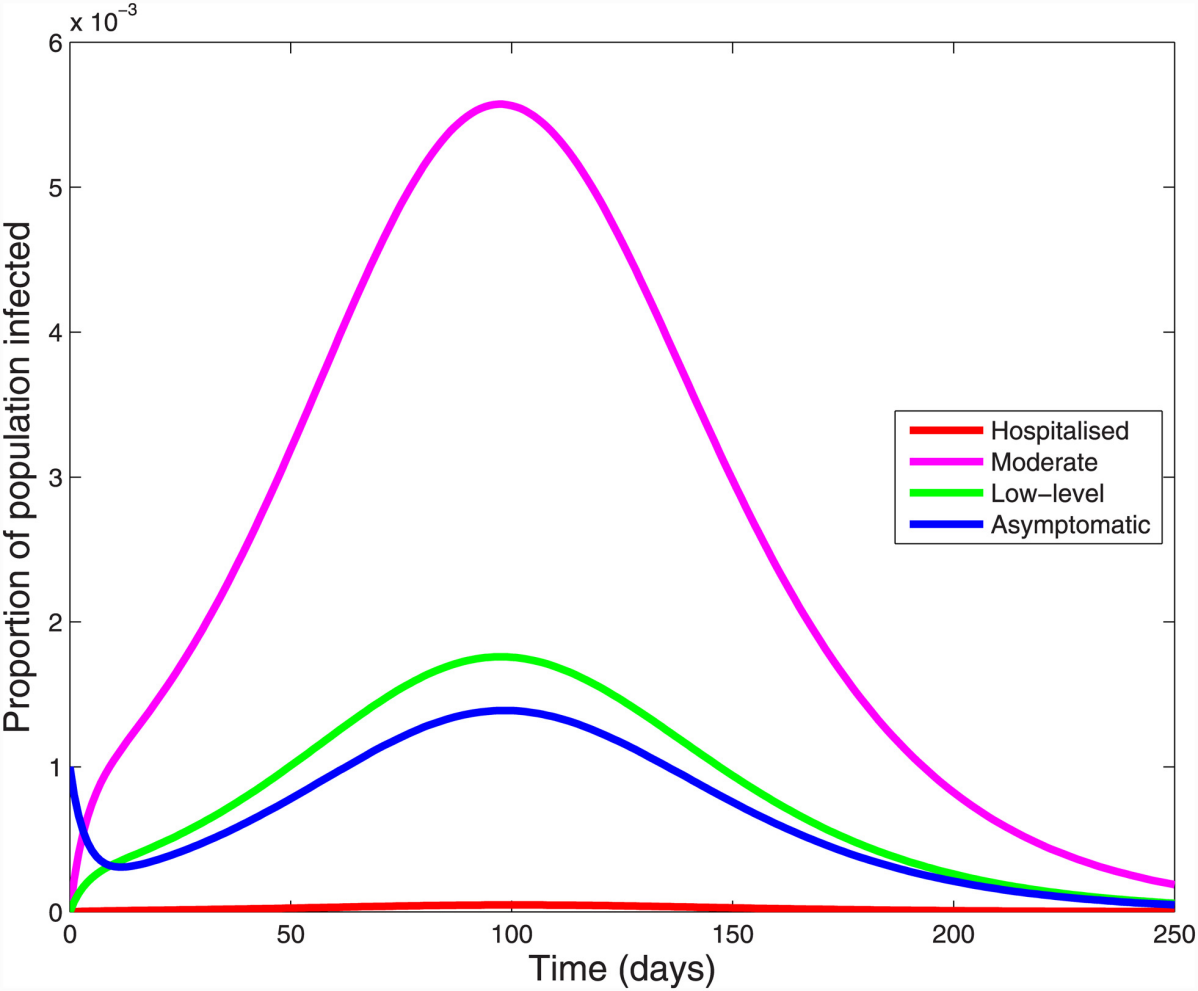
Incidence of influenza A(H1N1)pdm09 over time by infection severity.

Using our baseline parameters, we calibrated the model to give an effective reproduction number of 1.14. This value is at the lower limit of the published range, but resulted in a cumulative infection risk of 24%, slightly higher than the age-standardized estimates of 19% and 21% for Australia and New Zealand respectively [23, 27]; using higher effective reproduction numbers modelled higher and unrealistic cumulative infection risks. Fig 2 shows the cumulative incidence of infection in each severity stratum, where asymptomatic infections contribute 8.5%, low-level symptoms 3.8%, moderate symptoms 11.9% and hospitalised patients 0.06% of the total 24.2%. Asymptomatic infections peaked first at 95 days, followed two days later by those with low-level and moderate symptoms, while hospitalised cases peaked at 100 days (Fig 3).

Effective reproduction numbers for each infection severity category are shown in Table 2. These are defined as the mean number of individuals infected by a single individual in that severity category in a fully susceptible population. Under the baseline parameter settings the severity group with low-level symptoms infection accounts for the greatest transmission (*R*_e(L)_ = 1.82) followed by the asymptomatic group (*R*_e(A)_ = 1.32). The effective reproduction numbers in the moderate symptoms and hospitalised groups were less than 1.

**Table 2:** Effective reproduction number by severity category and parameter values.

Infection severity	Baseline *R*_e_	*R*_e_ after parameter adjustment from baseline					
		*p*_A_ = 0.13	*μ*_L_ = 0.7	*μ*_M_ = 0.6	*q* = 0.42	*γ*_M_ = 1/5.9	Susceptibility adjustment^
Asymptomatic	**1.32**	1.39	1.39	1.12	1.10	1.23	1.32
Low-level symptoms	**1.82**	1.92	1.49	1.55	1.52	1.70	1.82
Moderate symptoms	**0.81**	0.85	0.85	1.03	0.68	0.91	0.81
Hospitalised	**0.34**	0.36	0.36	0.29	0.29	0.32	0.32

^ Susceptibility for hospitalised = 0.89; moderate symptoms = 0.94; low-level symptoms = 0.94; asymptomatic = 0.94.

**Fig 4:**
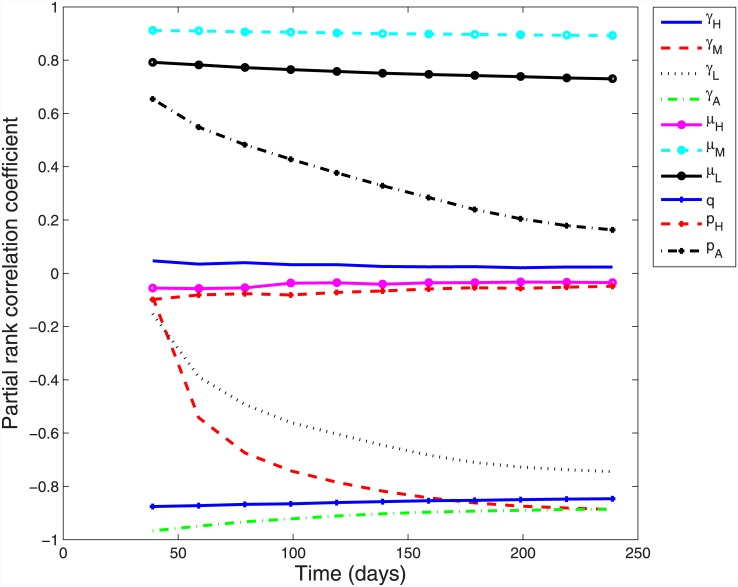
Partial rank correlation for infection severity proportion, mixing and recovery rate parameters over time.

The parameter uncertainty analysis showed that none of the hospitalised severity category parameters (proportion, mixing or recovery rate) had a discernible impact on the infection risk, with PRCC values near zero (Fig 4). The mixing (*μ*) parameters for low-level and moderate symptoms were strongly and positively correlated with infection risk, particularly moderate symptoms for which the final PRCC = 0.89. With PRCC values of −0.88 and −0.89 respectively, the recovery rate parameter for asymptomatic infection (*γ*_A_) and that for moderate symptoms (*γ*_M_) were strongly and negatively correlated with infection risk. The recovery rate for low-level symptoms was less important, but like the recovery rate for moderate symptoms, increased in importance from negligible levels at the start of the epidemic period. The importance of the proportion of asymptomatic infections also varied over the course of the epidemic, initially moderately and positively correlated with infection but declining to near neutrality by the end. However, ILI resulting in inability to undertake normal duties for two or more days (notated as *q* and a proxy for the proportion with moderate symptoms) was very important throughout the epidemic with PRCC = −0.85.

Variation of important model parameters, as identified by PRCC analysis, generally resulted in little difference in the broad trends observed from baseline values (Table 2). Increasing the perturbation of the parameters in the PRCC analysis to 20% showed very similar output, while at 40% the output remained qualitatively similar. The most marked change in the stratum-specific reproduction numbers occurred with raising the moderate symptoms mixing co-efficient from 0.4 to 0.6. Although this resulted in an effective reproduction number slightly greater than one for the moderate symptoms group, the reproduction number for the low-level symptoms group was still the greatest. Decreasing the *q* parameter (the proportion of symptomatic cases unable to undertake normal duties for two or more days) from 0.76 to 0.42 resulted in decreases in the effective reproduction number for all infection severity strata. The effect of lowering the proportion of asymptomatic cases from 0.35 to 0.13 had a relatively minor effect on stratum-specific reproduction numbers. There was no effect from application of susceptibility adjustments to each stratum. Importantly, under all alternative scenarios the effective reproduction numbers for the asymptomatic and low-level symptoms groups were greater than one and higher than those for the moderate symptoms and hospitalised groups.

## Discussion

Using a simple deterministic mathematical model, we show that transmission during the first wave of influenza A(H1N1)pdm09 was primarily driven by those with low-level symptoms (broadly defined as symptoms resulting in inability to undertake normal duties for at most one day) and, to a lesser extent, asymptomatic infections. Given such infections do not necessitate medical attendance (except perhaps for a certificate of absence) and individuals with mild symptoms are very unlikely to be tested, they would mostly be undetected by the health system. In contrast, infections resulting in moderate symptoms (inability to undertake normal duties for two or more days) or hospitalisation that are generally detected by the health system both had effective reproduction numbers less than one and a comparatively minor role in influenza A(H1N1)pdm09 transmission.

Development of the model necessitated a number of important assumptions, particularly with respect to baseline parameter values. Whilst most parameter values were sourced directly from the published literature, the relative mixing coefficients (*μ*) of each infection severity category were based on data on health-seeking behaviour, together with plausible assumptions concerning the behaviour of each category. The mixing coefficients for the low-level and moderate symptoms infection severity category in particular were influential model parameters. Nevertheless, sensitivity analyses using more conservative estimates of mixing coefficients were still broadly consistent with the baseline observation that asymptomatic and low-level symptoms infections were the most important drivers of transmission. Indeed, whilst reducing the mixing coefficient resulted in a lower effective reproduction number for the low-level symptoms group, this also resulted in an increase in transmission from those with asymptomatic infections. Furthermore, adjustment of the model for reduced susceptibility among the hospitalised group, who were more likely to be older and have pre-existing immunity, did not influence the main findings. The model does not account for possible higher levels of mixing in hospitalised patients prior to hospitalisation, although any effect is likely to be minimal given the low proportion of such cases.

Searches of the literature also identified heterogeneity in other parameter values, in particular the proportion of asymptomatic cases. The baseline value was set at 0.35 based on several transmission studies from Hong Kong, China and the USA [18, 20, 21], and comparable to estimates of asymptomatic infection for seasonal type A/H1N1, A/H3N2 and type B influenza of 31–38% [34]. At the lower end of the reported range were three studies with a reported asymptomatic proportion of 10–17% [15, 17, 30], but using an average of 13% in a sensitivity analysis had little effect on the infection severity stratum-specific reproduction numbers, as anticipated from the PRCC analysis. Other retrospective serological studies conducted in New Zealand [23], Austria [35] and a USA marine and naval cohort [36] indicated proportions of asymptomatic infections to be 45%, 84% and 53% respectively and were likely affected by recall bias and therefore not assessed in the sensitivity analysis.

With the exception of infections resulting in hospitalisation, the recovery rate parameters for all infection severity categories were important components of the model. Whilst these values were calculated from a systematic review of influenza A(H1N1)pdm09 virus shedding [25], they are also couched with some uncertainty. Firstly, the model assumes that the degree of infectiousness remains constant throughout the duration of viral shedding, and whilst there is some evidence that infectiousness wanes over this period, viral titres are highly variable and difficult to quantify [14–17, 20, 37, 38]. Secondly, most viral shedding studies used reverse transcriptase polymerase chain reaction (RT-PCR) to detect virus, which cannot differentiate between viable and non-viable virus and thus may overestimate the duration of viral shedding. However, this is likely to be at least partially offset (among those with symptomatic infections) by pre-symptomatic shedding. Pre-symptomatic influenza A(H1N1)pdm09 virus shedding has been reported for as long as three days before onset in less than 5% of cases [15, 39], although our model has not incorporated these data.

Several other limitations should be considered when interpreting the findings of this study. Due to scarcity of published data on absenteeism as a result of laboratory confirmed influenza, the division of symptomatic infections into those manifesting with low-level and moderate symptoms was based on data on days unable to undertake normal duties because of ILI. Whilst ILI is a non-specific outcome and will likely incorporate upper respiratory tract infections that are generally considered to be milder than influenza (which also frequently causes lower respiratory or systemic symptoms [40]), the positive predictive value of the syndromic ILI definition for influenza is likely to be relatively high because the data were collected during the peak of the first pandemic wave [24]. Nevertheless, testing of a wide range of the proportions with moderate and low-level symptoms in the sensitivity analysis showed the same relative differences between the effective reproduction numbers of each infection severity stratum. Finally, the model was developed and should be interpreted in the context of the first in-season wave of influenza A(H1N1)pdm09 in Australia. Estimating the relative importance of different levels of disease severity in influenza A(H1N1)pdm09 transmission in the northern hemisphere, subsequent pandemic waves in the southern hemisphere and seasonal influenza (that the pandemic strain has since become) would require the incorporation of immunity (either from prior infection or vaccination) and age group stratification into the baseline model.

### Public health implications

Our finding that low-grade and asymptomatic infections were the drivers of the first influenza A(H1N1)pdm09 wave in Australia helps explain why community transmission was apparently already well-established by the time influenza A(H1N1)pdm09 was detected. Furthermore, that transmission was being driven by those essentially invisible to the health system suggests that case-based pandemic control strategies such as antiviral distribution may not always be very effective. Whilst population-based interventions such as school closures may be more likely to be effective in interrupting transmission, such measures will probably be unnecessary when such a high proportion of infections are relatively mild. Public health plans and responses to pandemics in the future need to accommodate this contingency.

## Supporting Information

S1 File Model differential equations. (DOCX)
