# Supplementary material for: Transmission of the First Influenza A(H1N1)pdm09 Pandemic Wave in Australia Was Driven by Undetected Infections: Pandemic Response Implications
Source: PLoS One. 2015 Dec 21;10(12):e0144331. doi: 10.1371/journal.pone.0144331 (PMC4687009; doi:10.1371/journal.pone.0144331)
Supplement: S1 File — (DOCX) [file pone.0144331.s001.docx]

**Differential equations**

The force of infection is given by:

*λ* = *β*_A_.I_A_ + *β*_L_.I_L_.+ *β*_M_.I_M_ + *β*_H_.I_H_

The differential equations that require solving are:

dS_A_/dt = −*λ*.S_A_

dS_L_/dt = −*λ*.S_L_

dS_M_/dt = −*λ*.S_M_

dS_H_/dt = −*λ*.S_H_

dI_A_/dt = *λ*.S_A_ − *γ*_A_.I_A_

dI_L_/dt = *λ*.S_L_ − *γ*_L_.I_L_

dI_M_/dt = *λ*.S_M_ − *γ*_M_.I_M_

dI_H_/dt = *λ*.S_H_ − *γ*_H_.I_H_

dR_A_/dt = *γ*_A_.I_A_

dR_L_/dt = *γ*_L_.I_L_

dR_M_/dt = *γ*_M_.I_M_

dR_H_/dt = *γ*_H_.I_H_

subject to the initial conditions S_A_(0) = 0.35 − I_A_(0), S_H_(0) = 0.0025, S_L_(0) = (1 − (S_A_ + S_H_)).(1 − *q*), S_M_(0) = (1 − (S_A_ + S_H_)).(*q*), I_A_(0) = 0.001, and I_L_(0) = I_M_(0) = I_H_(0) = R_A_ = R_L_(0) = R_M_(0) = R_H_(0) = 0.
